# The Natural History of T Cell Metabolism

**DOI:** 10.3390/ijms22136779

**Published:** 2021-06-24

**Authors:** Michel Y. Braun

**Affiliations:** Institute for Medical Immunology (IMI), Faculty of Medicine, Université Libre de Bruxelles (ULB), 6041 Gosselies, Belgium; michel.braun@ulb.be

**Keywords:** T lymphocytes, metabolism, activation, memory, Treg

## Abstract

The cells of the immune system, particularly the T lymphocytes, have two main features that distinguish them from the cells of other tissues. They proliferate after activation and have the ability to move in tissues and organs. These characteristics compel them to develop metabolic plasticity in order to fulfil their immune function. This review focuses on the different known mechanisms that allow T cells to adapt their metabolism to the real-life circumstances they operate in, whether it is to exit quiescence, to differentiate into effector cells, or to participate in immune memory formation. Some of the metabolic adaptations to environmental variations that T cells are likely to undergo in their immune monitoring function are also discussed.

## 1. Introduction

The idea of using the immune system to eradicate tumours is well-established. However, the use of therapies based on vaccination to control tumour growth has shown, until now, limited efficacy. This situation is thought to be a direct consequence of the ability of the tumour microenvironment to alter, both directly and indirectly, the functions of immune cells. This is how tumour cells express inhibitory immune receptor ligands that suppress T cell function. Therapy targeting one of these inhibitors, the programmed cell death protein 1 (PD-1) and its ligand PD-L1, as well as monoclonal antibodies blocking the interaction between cytotoxic lymphocyte antigen 4 (CTLA-4) and its ligands CD80 and CD86, have brought great hope in the fight against solid or haematological malignancies [1,2]. In addition to their ability to directly inhibit T cells, tumours, due to their high metabolism, create a harmful metabolic environment preventing the successful development of adaptive anti-tumour immune responses. For example, their high consumption of nutrients depletes the medium in resources necessary for the survival of T lymphocytes in the tumour [3]. Likewise, the accumulation of metabolic waste, such as lactate or kynurenine, inhibits the activity of T lymphocytes [4,5]. Finally, tumours have been described as a metabolically favourable environment for the development of T regulatory lymphocytes (Tregs) which suppress the activity of tumour-specific effector T cells [6,7]. Understanding how T cells are able to adapt their metabolism to the changing conditions in which they operate is of critical importance for the development of effective immune therapies in an extensive variety of diseases, and more specifically, against cancer.

## 2. Early T Cell Metabolism

**Figure 1:**
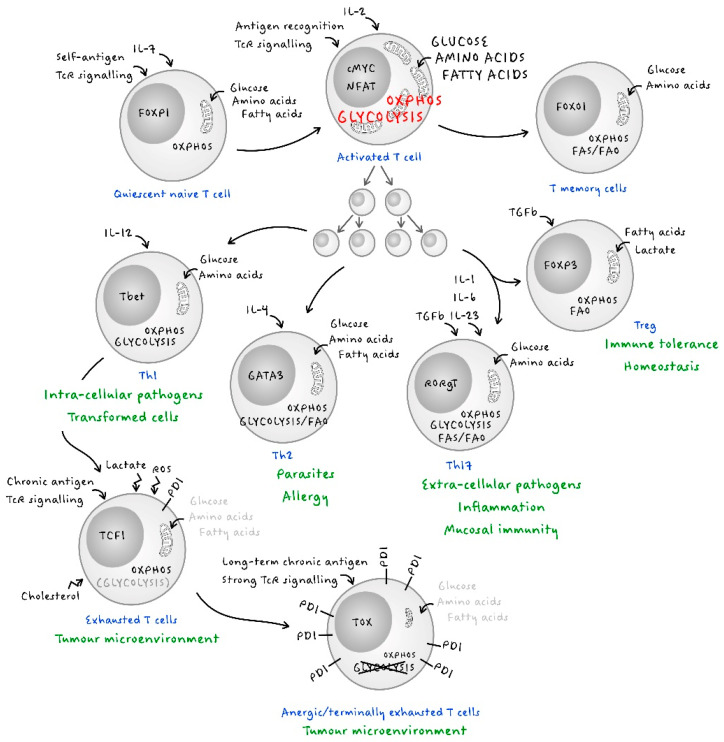
The natural history of T cell metabolism. Throughout their life, T cells must adapt their metabolism to the different circumstances they encounter, whether it is according to the environment in which they evolve, the type of pathogens they fight, or the function they perform.

Mature T cells arise from the thymus where they have differentiated into immunocompetent cells to circulate throughout the body, surveying for infection. As recent thymic emigrants, they acquire a low basic metabolic capacity by mechanisms likely involving autophagy of their mitochondria [8]. In fact, they adopt a metabolism that allows them to survive for long periods of time while waiting to encounter the antigen for which they rearranged a specific T cell receptor (TCR). During this quiescent period, they require only basal replacement biosynthesis and rely on low amounts of glucose and fatty acids to fuel oxidative phosphorylation (OXPHOS) (Figure 1) [9]. The survival of quiescent naive T cells is maintained by weak TCR signalling produced by the recognition of self-peptides presented on self-MHC molecules [10,11]. It also relies on the secretion of specific cytokines. T cells that fail to receive normal microenvironmental clues, such as signalling from cytokine receptors, undergo cellular atrophy with decreased cell size and metabolism that ultimately leads to apoptosis [12]. IL-7 is expressed by stromal cells in T cell zones [13] and is critical for the survival of quiescent T cells [14,15]. One of the roles played by IL-7R expressed at the surface of quiescent T cells is to promote basal metabolism required to support T cell survival [9], and functional studies have validated *Il7ra* as a target gene of the Forkhead-box transcription factor FOXO1, a transcription factor essential for the maintenance of naive T cells. [16].

Another key regulator responsible for the maintenance of quiescence in naive T cells is tuberous sclerosis 1 (TSC1) [17]. This molecule is known for its control of the mechanistic target of rapamycin (mTOR) which is responsible for the metabolic changes triggered by activation in T cells. TSC1 regulates the activity of mTORC1 and mTORC2, and mTORC1 activation is essential for the disruption of immune quiescence and homeostasis. TSC1 deficiency leads to the disrupted homeostasis of peripheral T cell populations by causing cell-autonomous loss of quiescence and hyperactive responses to TCR stimulation [17]. This results in excessive apoptosis of T cells via the Bcl-2 family–dependent intrinsic apoptotic pathway. In addition to TSC1, signalling involving the sphingosine-1-phosphate receptor 1 (S1P1), is essential for naive T cell survival [18]. The chemoattractant sphingosine 1-phosphate (S1P) guides T cell circulation among secondary lymphoid organs in their search for antigen. Lymphatic endothelial cells support the survival of T cells by secreting S1P via the sphingolipid transporter 2 (SPNS2). S1P signals through S1P1 on T cells and maintains the mitochondrial content of naive T cells, providing cells with the energy needed to continue their constant migration. Given the energy cost of constant migration to sample antigen, the use of the same signalling to direct circulation and to promote mitochondrial function could be an efficient way for naive T cells to supply the fuel they require.

When a T cell encounters its antigenic peptide presented by MHC molecules (pMHC) expressed at the surface of antigen-presenting cells (APC), the high affinity of its TCRs for the pMHC complexes causes TCR clustering and the formation of an immune synapse between the T cell and the APC. TCR signals induce the activation of the nuclear factor c-MYC which is required for the expression of the global metabolic transcriptome that drives metabolic reprogramming in activated T lymphocytes (Figure 1) [19]. In addition, TCR signals, together with co-stimulation, stimulate the global metabolism of the cell, a process under the control of phosphoinositide-3 kinase (PI3K)/AKT/mTOR pathway [20] which coordinates several metabolic programs in T cells including glycolysis, lipid synthesis, and oxidative phosphorylation. The divalent cation Ca^2+^, a vital second messenger for TCR signalling in T lymphocytes, modulates metabolic changes that occur after activation [21]. Calcium entry from cellular stores is the main Ca^2+^ source that regulates Ca^2+^-dependent signalling molecules, such as calcineurin, and controls cell cycle entry of quiescent T cells. Upon antigen recognition, Ca^2+^ entry was shown to direct the metabolic reprogramming of naive T cells by regulating the expression of glucose transporters, glycolytic enzymes, and metabolic regulators through the activation of the nuclear factor of activated T cells (NFAT) and the PI3K-AKT kinase-mTOR pathway [21].

To come out of the quiescent state demands a lot of cellular energy and requires the significant commitment of the mitochondrial metabolism. Mitochondrial ATP production early after T cell activation is indeed largely increased and is essential for exiting quiescence. Raptor-mTORC1, a central regulator of T cell quiescence exit [20], controls the activation of two mitochondrial pathways that are particularly necessary, namely the mitoribosomes which lead to the translation of mitochondria-encoded electron transport components to support OXPHOS, and complex IV (cytochrome c oxidase) which is required to define the proton electrochemical potential on which the ATP synthase relies on to produce ATP [15]. These elements are supported by the observation that T cells lacking COX10, a critical regulator of complex IV, are unable to exit quiescence [15,22]. Early studies indicated that, in contrast to the necessity of high levels of OXPHOS, glycolysis seemed to be dispensable, since limiting the glycolytic activity by induced deficiency of the limiting enzyme hexokinase-2 (HK2) or stimulating T cells in glucose-free galactose-containing medium (which forces T cells to use OXPHOS), did not prevent T cell quiescence exit [15,23]. However, a more recent study appears to indicate that early glycolysis engagement after T cell activation is required for acute cytokine production [24]. Additionally, gene transcription activity is very limited in the early steps of T cell activation, suggesting that expression of the metabolic proteome at this stage is mostly regulated post-transcriptionally at the protein level by mechanisms metabolically less costly than protein synthesis from genomic DNA [15,25].

Reactive oxygen species (ROS) generated in the mitochondria act as signalling messengers for the activation of T cells. Indeed, CD4^+^ T cells lacking the complex III subunit UQCRFS1 required for ROS production fail to activate NFAT–dependent transcription and to produce IL-2 [26]. On the other hand, a more recent study has also highlighted the importance of ROS inhibition by the antioxidant glutathione since failure to scavenge accumulating ROS led to decreased mTOR and cMYC activities and a subsequent block in T cell activation [27]. Taken together, these observations show that fine control of ROS is required during T cell activation.

Another early metabolic event that precedes activation-driven cell division in T cells is the mitochondrial processing of serine, a non-essential amino acid. Haigis and co-workers reported that naive CD4^+^ T cell activation induces a unique program of mitochondrial biogenesis [28]. Using mass spectrometry, they identified substantial remodelling of the mitochondrial proteome over the first 24 h of T cell activation, giving rise to mitochondria with a distinct metabolic signature where rapidly increased processing of serine into one-carbon metabolites, enables nucleotide synthesis for T cell proliferation and survival.

## 3. Effector Metabolism in T Cells

One of the hallmarks of activated T cells is the increased expression of nutrient transporters [29] and enzymes involved in glycolysis and mitochondrial metabolism for ATP production and biosynthesis [19,20,29]. The main metabolic signatures of T cell proliferation in vitro are increased glucose uptake and lactate production commonly referred to as the Warburg effect, which takes place 24 h after activation. Blocking glycolysis also reduces effector function in T cells [23,30,31,32], highlighting the importance of metabolic reprogramming and nutrient availability for proper immune function. Our current understanding of T cell metabolism has been made possible by in vitro ^13^carbon-based isotopic labelling techniques that facilitate metabolic tracking of nutrients in cells [33]. These have allowed the identification of metabolic pathways necessary for the function and expansion of T cells, in particular glucose and glutamine [23,34,35]. One of the limitations of these techniques applied in vitro is that immune cells are grown at optimal nutrient and oxygen concentrations, often different from those observed in vivo [36]. In addition, injection of ^13^carbon-labeled metabolites in mice and humans also demonstrated differences in nutrient utilisation by stimulated T cells in vivo compared to cultured cells [37,38,39,40,41]. Finally, T cells exhibit metabolic plasticity when the available amounts of nutrients such as glucose are limited [35,42,43], illustrating that the nutrients present in the direct environment influence T cell metabolism. These observations also suggest that T cells compete with surrounding tissues for nutrients necessary for their growth and proliferation [3,42]. It should also be noted that oxygen tensions in inflamed tissues are generally low. The molecular mechanisms that ensure adaptation to hypoxia are operational in immune cells, thus enabling immune surveillance in all tissue microenvironments and preventing “safe shelters” for pathogens. Activated T cells adapt to changing energy supplies in hypoxic areas of inflamed tissue by using the hypoxia-inducible factor 1 (HIF1) to switch to glycolysis as the primary energy source [44].

The diversion of pyruvate to lactate requires the ability to control the amount of lactate present in the cytoplasm or to deal with the risk of feedback inhibition of glycolytic activity [45]. Pharmacological inhibition of MCT1, a monocarboxylate transporter responsible for lactate export, has been shown to inhibit T cell proliferation [46]. In experiments involving the specific deletion of MCT1-coding gene *Slc16a1* in T cells, plasma lactate accumulation correlated with the inhibition of glucose–dependent nucleotide synthesis, illustrating the importance of the evacuation of lactate from T cells that exhibit intense glycolytic activity [MYB, unpublished]. Interestingly, lactate was not the only metabolite that accumulated in activated MCT1-deficient T cells. A significant accumulation of TCA intermediates citrate and α-ketoglutarate was also observed after stimulation [MYB, unpublished]. This observation would imply that, in the absence of MCT1 expression, glucose-derived pyruvate is preferably oxidised in T cells, but that oxidation is stopped at the step that leads to accumulation of α-ketoglutarate. α-Ketoglutarate is known to serve as an obligate substrate for a subset of chromatin-modifying enzymes [47,48,49] and was recently identified to link tumour suppression to the transcriptional program enforced by the tumour suppressor factor p53 [50]. Whether accumulation of glucose-derived α-ketoglutarate regulates proliferation in MCT1-deficient T cells is an attractive hypothesis that deserves attention in future experiments. Another example of epigenetic regulation of T cell function linked to mitochondria metabolism has been identified by studying T cells carrying a null mutation for the gene of lactate dehydrogenase A (LDHA) [51]. LDHA maintains high concentrations of acetyl-coenzyme A to enhance histone acetylation and gene transcription. Ablation of LDHA in T cells causes an increase in OXPHOS after activation and a subsequent drop in acetyl-Co A-mediated acetylation of histones bound to the promoter and enhancer of the *Ifng* gene, preventing transcription and production of IFN-γ. Taken together, these studies highlight the crucial role played by cell metabolism in controlling gene expression during T cell activation.

With activation, biosynthesis increases drastically in T lymphocytes, to the point that it must be slowed down to avoid too great a drop in cellular ATP. This regulation is believed to be the direct consequence of the activity of AMP-activated protein kinase (AMPK) and aims to ensure a sufficient amount of cellular energy for the proliferation and function of T lymphocytes [52,53]. Activation of AMPK decreases fatty acid and protein synthesis and promotes fatty acid oxidation (FAO) and mitochondrial biogenesis. AMPK also up-regulates the rate of glycolysis through phosphorylation of 6-phosphofructo-2-kinase/fructose-2,6-biphosphatase isoform 3 (PFKFB3) and increases glucose uptake [54]. Moreover, AMPK recently emerged as an important sensor and regulator of mitochondrial ROS and a regulator of ROS neutralisation by antioxidant cellular systems, thereby maintaining adequate cellular oxidative balance [55]. In a recent study, AMPK was shown to be dispensable for short-term proliferation but was required for sustained long-term T cell proliferation and effector/memory T cell survival [56]. Mechanistically, AMPK promoted the accumulation of effector/memory T cells by enhancing the mitochondrial membrane potential of T cells, thereby limiting ROS production.

The adaptive immune response is orchestrated by different subsets of CD4^+^ T lymphocytes, each subset promoting immunity through different effector arms. They all appear to be glycolytic in nature during the expansion phase that follows activation but adopt different metabolic profiles for maintenance and function (Figure 1). Th1 lymphocytes, which promote immune responses against viruses and intracellular bacteria, also appear to require glycolysis for their function [23,51]. It has been proposed that aerobic glycolysis enhances IFNγ production by sequestering GAPDH away from binding to the AU-rich elements within the 3′ UTR of IFNγ mRNA, thereby preventing inhibition of its translation [23]. As mentioned before, another study has shown that LDHA activity maintains a high concentration of acetyl-CoA to enhance histone acetylation and the transcription of IFNγ [51]. Thus, aerobic glycolysis is a metabolically regulated signalling mechanism needed to control cellular function in Th1 cells. Th2 CD4^+^ T cells, which are triggered by parasite infections and allergic reactions, predominantly undergo aerobic glycolysis in order to proliferate and perform their effector functions, just like the Th1 cells [57]. However, studies have pointed out that some additional metabolic programming occurs when Th2 cells enter inflamed tissue sites and tissue-migrated Th2 cells display lipid-based metabolism as their prominent feature [58]. Single-cell RNA sequencing of the T cell response to house dust mites revealed that pathogenic airway Th2 cells are highly enriched for genes associated with lipid metabolism [58]. Moreover, using inhibitors of lipid metabolism induced a drastic reduction in pathologies mediated by Th2 lymphocytes [57]. The survival of tissue-resident memory T cells requires exogenous lipid uptake and metabolism [59]. Determining whether increased reliance on lipid metabolism is a strategy to prolong Th2 cell longevity in tissues would be of significant interest.

Th17 lymphocytes specialise in immune defence against extracellular bacteria. Although glycolysis is also essential for their development, the environmental context in which T cells thrive can modulate the metabolic pathway differently. It has been shown that whereas CRISPR-mediated targeting of glycolysis in T cells in mice results in a global loss of Th17 cells, the single deficiency of the glycolytic enzyme glucose phosphate isomerase (GPI) selectively eliminates inflammatory encephalitogenic and colitogenic Th17 cells, without substantially affecting homeostatic microbiota-specific Th17 cells [60]. In homeostatic Th17 cells, the partial blockade of glycolysis upon GPI inactivation is compensated by pentose phosphate pathway flux and increased mitochondrial respiration [60]. The need to enhance glucose metabolism and engage glycolysis for Th17 differentiation is also illustrated by the inability to generate Th17 cells in the absence of the nuclear factor HIF1α [61,62,63]. HIF1α promotes increased glucose uptake via the up-regulation of GLUT1 and reinforces glycolysis through the up-regulation of pyruvate dehydrogenase kinase 1 (PDK1) [64]. Increased PDK1 activity, in turn, prevents entry of pyruvate into the TCA cycle and redirects it to be metabolised into lactate. HIF1α has been demonstrated to be a critical factor in the development of Th17 cells versus inducible T regulatory cells (Tregs) and the pro-inflammatory cytokine IL-6 is required for inducing HIF1α expression [65]. Fatty acid synthesis (FAS) has also been shown to be essential for Th17 development. Unlike Tregs, which use exogenous fatty acids (see below Section 5), Th17 cells primarily depend on de-novo FAS [66]. Inhibition acetyl-CoA carboxylase 1 (ACC1), which is crucial for FAS, impairs the generation of human and mouse Th17 cells but favours the development of Tregs. Moreover, T cell-specific deletion of ACC1 in mice, or in vivo treatment with specific inhibitors, has been shown to attenuate Th17 cell-mediated autoimmune disease [66].

## 4. The Metabolism of Adaptive Immune Memory

Memory T cells were first identified as a T cell population that was found to survive long after the infection had cleared [67]. This memory population was shown to have an increased ability to restrict viral replication upon secondary infection, and thus indicated the importance and potency of memory T cells compared to naive or effector T cells [67]. In addiction to prolonged survival, migrating is another defining feature of classical memory T cells. Central memory T cells (Tcm) and effector T memory cells (Tem) are defined in blood based on their expression CC-chemokine receptor 7 (CCR7) [68]. CCR7 mediates homing of T cells to secondary lymphoid organs via high endothelial venules (HEV). Therefore, CCR7^-^ Tem have the capacity to migrate and reside into tissues whereas CCR7^+^ Tcm are found mostly in secondary lymphoid organs. However, subsequent experiments have clearly indicated the strict resident nature of a substantial proportion of memory CD8^+^ T cells found in tissues, qualified as “tissue resident” memory T cells (Trm). [69,70,71]. The precise location of Trm within tissues gives the host enhanced regional immunity that can be activated upon subsequent local infections. It is also known that some memory T cells reacquire the ability to undergo long-term self-renewal [72,73]. These cells, termed “T memory stem cells” (Tscm), representing approximately 2–4% of all circulating T lymphocytes, seem to be extremely durable, and can rapidly differentiate into more mature central memory, effector memory, and effector T cells.

Several studies indicate that memory T lymphocytes depend mainly on lipid catabolism to meet their long-term survival needs [59,74,75,76]. While FAS is important for T cell proliferation and differentiation into effector cells, a metabolic shift towards FAO appears to be necessary for memory T cell survival. The observation that memory T cells use intrinsic cellular lipolysis to mobilise fatty acids for FAO, suggests that in these cells lipids must be synthesised before mitochondrial oxidation can occur [76]. This conclusion is also supported by Lee et al., who showed that antigen-specific CD8^+^ T cells deficient for the expression of ACC1 (responsible for the production of malonyl CoA used in the synthesis of long-chain fatty acids) were characterised by impaired peripheral persistence and homeostatic proliferation after immunisation [77].

Mitochondrial respiration can be supported by different carbon sources, such as glucose pyruvate, glutamine, and other amino acids and lipids, including not only long-chain fatty acids but also medium and short-chain fatty acids. This last route of oxidative energy supply was recently highlighted by the observation that CPTA1 was dispensable, for the formation of memory T lymphocytes [78]. Unlike long-chain fatty acids, short fatty acids have the advantage that they do not require active transport and can migrate freely through bilipidic barriers such as cytoplasmic and mitochondrial membranes. This is how microbiota-derived short-chain fatty acid butyrate is believed to enhance the memory potential of activated CD8^+^ T cells and is required for optimal recall responses upon antigen re-encounter [79]. These findings also suggest that, in addition to fuelling lipid oxidation, microbial metabolites could guide the metabolic rewiring of activated CD8^+^ T cells to enable memory transition.

Mitochondria are metabolic organelles capable of actively transforming their ultrastructure. CD8^+^ effector T cells possess punctate mitochondria, while memory CD8^+^ T cells maintain fused mitochondrial networks [80]. The mitochondrial dynamin-like GTPase OPA1 is known to promote inner mitochondrial membrane fusion and is essential for the maintenance of mitochondrial cristae as well as for the stability of electron transport chain complexes [81]. Thus, the expression of OPA1 is necessary for memory T cells, but not for effector T cells. Likewise, forced expression of OPA1 in effector T cells imposes characteristics of memory cells and enhances the anti-tumour function [81]. These data suggest that mitochondrial fusion in memory T cells configures the complex associations of the electron transport chain promoting OXPHOS and FAO, while the fission of mitochondria in effector T cells reduces OXPHOS and promotes aerobic glycolysis necessary for antigen-induced proliferation [82].

Compared to naive and effector T cells, memory T cells were shown to have increased mitochondrial mass and greater spare respiratory capacity (SRC) [83]. This would allow for the rapid generation of mitochondrial ATP upon activation and would give a bioenergetic advantage upon secondary antigen stimulation. IL-15, a cytokine important for the maintenance of memory T cells, was shown to regulate SRC and oxidative metabolism by promoting both mitochondrial biogenesis and the expression of carnitine palmitoyl transferase (CPT1a), a metabolic enzyme involved in long-chain fatty acid transport, such as palmitoyl-CoA, to the inter-membrane space of mitochondria for FAO [83]. However, a recent report has challenged this concept by providing evidence that antigen-experienced memory T cells, as defined by their expression of CD44, do not express the IL-15 receptor (IL-15R) and would not exhibit increased SRC [84]. According to this study, IL-15R and increased SRC would characterise CD44^+^ CD49d^low^ “virtual memory” or “memory-like” CD8^+^ T cells [84]. These cells arise in the absence of antigenic exposure upon type one interferon signalling and express high levels of the nuclear factor Eomesodermin (EOMES) [85]. Their increased SRC appears to be a more relevant indicator of their extended longevity rather than any particular function [84]. If high SRC is indeed required for cell survival, it would be informative to assess SRC in other long-lived T cell subsets, such as TSCM, which are highly proliferative, and TRM, which retain a level of proliferative capacity in situ and are highly IL-15 dependent [86,87].

## 5. T Regulatory Cells

T regulatory cells (Tregs) express the transcription factor FOXP3 and suppress immune responses by competing for IL-2, a growth hormone required for the expansion of activated conventional T cells, by producing inhibitory cytokines, such as IL-10 and TGF-β, and by checkpoint mechanisms (PD-1, CTLA4, LAG3) [88,89]. The activity of these T cells is important for the resolution of immune responses and the maintenance of immune homeostasis in a number of organs. Their unique function in immune responses makes them dependent on a specific metabolic activity for their development and function. Michaleck and colleagues showed that although Th1 and Th17 cells (which express high amounts of the glucose transporter GLUT1) are highly glycolytic, Treg cells generated through in vitro polarisation of CD4^+^ T cells preferentially use lipid oxidation (Figure 1) [90,91]. However, those differentiating conditions were induced in vitro by the addition of high doses of cytokines (TGFβ). TGFβ signals drive Treg differentiation and activates AMPK which promotes FAO and deviates Th17 cells towards a Treg phenotype [89,92]. These in vitro studies seemed to indicate that, unlike the T effector cells, the Tregs favoured OXPHOS and FAO for their development and function (Figure 1). It was therefore very surprising to observe that un-manipulated freshly-isolated human Tregs were found to be highly glycolytic at both the proteomic and the functional levels when compared to ex vivo-isolated conventional T cells [93]. Since glycolysis is known to promote biosynthesis for rapid T cell growth, this observation confirmed that Treg cells actively proliferate in vivo both in humans and in mice [94,95]. Ex vivo Tregs are also metabolically more active than conventional T cells, expressing a higher level of mTOR, and mTORC1 signalling is a pivotal positive determinant of the Tregs function in mice [96,97]. Moreover, it has been shown that glycolysis is important for the generation of inducible Treg (iTreg) cells [98].

The various observations which describe the Tregs to rely either on glycolysis or on FAO for their energy needs suggest a complex relationship between metabolism and function in these cells. One study offers a possibility that reconciles these seemingly opposing situations [99]. It was observed that signals that promote Treg cell proliferation increased PI(3)K-AKT-mTORC1 signalling and glycolysis. Alternatively, the transcription factor FOXP3 opposed PI(3)K-AKT-mTORC1 signalling to limit glycolysis and anabolic metabolism while increasing lipid and oxidative metabolism [99]. Moreover, whereas glycolysis induced Treg proliferation, this was inversely proportional to their suppressive capacity. The utilisation of OXPHOS, on the contrary, was required for maximal suppressive activity. Thus, the Tregs would adopt a different energy profile depending on whether they are expanding or performing their function. The observation that control of PI(3)K signalling by the phosphatase PTEN in Treg cells is critical for maintaining their function and stability supports this concept [100]. It remains to be seen whether this metabolic dichotomy related to proliferative capacity or function is always mutually exclusive or whether there are exceptions. The observation that commensal microbe-derived butyrate induces the differentiation of colonic Tregs suggests that this might depend on their direct environment [101,102].

The fact that Tregs are present in a wide variety of organs and tissues suggests that they must indeed be able to adapt to different metabolic situations. The best example of this is the ability of Tregs to adapt to the harsh metabolic environment imposed inside solid tumours. Glucose consumption by tumours metabolically restricts T cells, resulting in decreased mTOR activity and glycolysis [3]. Likewise, the excess lactate produced by the glycolytic activity of tumour cells can regulate the activity of tumour-infiltrating T cells (TIL) [5,103,104]. In Tregs, the transcription factor FOXP3 was shown to reprogram metabolism by suppressing Myc and glycolysis, enhancing oxidative phosphorylation, and increasing nicotinamide adenine dinucleotide (NAD) oxidation [6]. This adaptation to lactate appears to be mediated by the specific expression of CD36 at the surface of intra-tumour Tregs [7]. CD36 via peroxisome proliferator-activated receptor-β (PPARβ) signalling sustains survival and function in intra-tumour Tregs by modulating mitochondrial fitness and NAD levels, which are critical for metabolising lactate [7].

## 6. The Metabolism of T Cell Exhaustion

Exhausted T cells (Tex) are distinct cell lineages that arise during chronic viral infections and cancers in animals and humans. Tex cells are characterised by progressive loss of effector functions maintained by inhibitory receptor expression and metabolic deregulation [105,106]. Exhaustion develops following strong TCR-mediated stimulation or during sustained chronic exposure to antigen and affects both CD4^+^ and CD8^+^ T cells [107,108,109]. Originally, Tex were described as having their effector function actively controlled by the engagement on their surface of inhibitory receptors, such as PD-1 [108]. Rafi Ahmed’s group was the first to demonstrate, in the mouse model of chronic LCMV infection, that Tex functional unresponsiveness could be lifted and the infection controlled by the administration of antibodies neutralising the interaction between PD-1 and its ligand [108]. More recently, John Wherry and his colleagues clearly demonstrated that the population of Tex is heterogeneous and can be divided into at least two sub-groups, based on the intensity of PD-1 expression and their expression of molecules such as CD69 [110]. This is how PD-1^int^ CD69^-^ Tex, whose activity can be restored by inhibition of the PD-1 checkpoint, are distinguished from PD-1^high^ CD69^+^ Tex which are terminally differentiated T cells and remain insensitive to anti-PD1 treatment (Figure 1). Several key pathways have been implicated in the establishment of Tex, including those mediated by the nuclear factors TCF1 and TOX [111,112]. 

The cytoplasmic domain of PD-1 has two tyrosines. The more proximal one constitutes an immunoreceptor tyrosine-based inhibitory motif (ITIM), whereas the distal residue forms an immunoreceptor tyrosine-based switch motif (ITSM). Upon stimulation, PD-1 becomes phosphorylated at its tyrosine-based motives which then bind the Src homology 2 (SH2) domains of SH2-containing phosphatase 2 (SHP2) [113]. PD-1 suppresses signalling through the TCR and T cell costimulatory receptor CD28 [113]. Thus, T cells must receive the PD-1 signal in conjunction with the antigen-specific TCR signal to negatively influence T cell function. Consequently, PD1 ligands, PD-L1 or PD-L2, and peptide/MHC must be co-expressed by the same antigen-presenting cell to signal through PD-1. In vitro, the cross-linking of PD-1 on the surface of T cells attenuates PI(3)K-AKT-mTORC1 signalling with direct impacts on metabolism, mainly the suppression of glycolysis [114,115]. Likewise, an in vivo blockade of PD-1 has been shown to restore anabolic metabolism and glycolysis in PD-1^int^ CD8^+^ Tex in an mTOR-dependent manner [3,116]. However, as mentioned above, long-term exposure to chronic antigen stimulation can lead to a deeper state of T cell unresponsiveness characterised by higher expression of inhibitory receptors [116,117]. In these terminally differentiated Tex, blocking PD-1 does not restore their glycolytic capacity but increases mitochondrial superoxide production and induces cell death by apoptosis [116,117].

In the context of solid tumours, TIL usually exhibits a state of functional exhaustion that limits their anti-tumour activity. Functional unresponsiveness can be the result of direct repressing signals from the tumour microenvironment [118] or competition for nutrients with tumour cells [3], but is also actively maintained by the engagement of inhibitory receptors, such as PD-1, present at the surface of TILs, by ligands expressed on cancer cells [2]. One of the main metabolic features of exhausted TILs is the persistent loss of mitochondrial mass and function [2,116,119]. In particular, TILs show loss of PPAR-gamma co-activator 1α (PGC1α) which program mitochondrial biogenesis [120]. Pharmacological activation of PGC1α has been shown to activate mitochondria in T cells and to up-regulate OXPHOS, resulting in improved immune function [121]. On the contrary, chronic antigenic T cell stimulation under hypoxic conditions promotes BLIMPS-1-mediated inhibition of PGC1α-dependent mitochondrial reprogramming [122]. The loss of mitochondrial function generates reactive oxygen species (ROS) to levels sufficient to promote exhaustion and lowering ROS production in T cells improves response to checkpoint immunotherapy [122,123]. Lymphocyte activation gene-3 (LAG-3) is an inhibitory receptor expressed by CD4^+^ and CD8^+^ T cells and in particular by TILs [124]. LAG-3 deficiency enhances the metabolic profile of CD4^+^ T cells by elevating levels of mitochondrial biogenesis [125]. In vivo, LAG-3 blockade partially restores expansion and the metabolic phenotype of wild-type CD4^+^ T cells to levels of LAG-3-deficient CD4^+^ T cells, demonstrating that LAG-3 controls these processes [125]. Whether LAG-3 limits mitochondria biogenesis in TILs, however, remains to be determined.

TILs are also compromised in glycolytic metabolism. The blockade of either PD-1 or CTLA-4 inhibitory receptors augments the glycolytic metabolism in TILs responding to mouse sarcoma tumours, demonstrating that glycolysis is the target of multiple inhibitory receptors [3]. In a recent study, CD8^+^ TILs were shown to have low levels of phosphoenolpyruvate (PEP) and enolase activity, implying that the PEP production step of glycolysis is the point at which glycolysis is disrupted in CD8^+^ TILs [126]. Combined inhibitory receptor blockade therapy recruited enolase-active CD8^+^ TILs into tumours, whereas targeting individual checkpoint molecules did not significantly improve enolase activity in CD8^+^ T cells [126]. Taken as a whole, these experiments demonstrate that the underlying basis of the dysfunction of TILs is both a limited OXPHOS and insufficient glycolysis to support effector functions despite their ability to effectively transport glucose. This indicates that CD8^+^ TILs represent a population of cells that undergo constant antigenic stimulation and that their decreased metabolic activity is not the consequence of immunological quiescence.

One of the major consequences T cells have to face when arriving in the tumour microenvironment is the competition for nutrients and in particular glucose deprivation. In addition to the impact on metabolic activity, the limitation of glucose can also have consequences on the epigenetic regulation of gene expression in TIL. Since the breakdown of glucose into pyruvate during glycolysis is the prime source of intracellular acetyl-CoA, glucose limitation might give a specific histone acetylation profile in TIL that could explain their exhausted phenotype [51]. This possibility is supported by the observation that acetate supplementation is sufficient to restore IFNγ production in T cells isolated from melanoma [43]. Glucose deprivation due to tumour cell growth has also another consequence for TILs, i.e., the release of important quantities of lactate, the by-product of glycolysis, in the tumour microenvironment. Lactate has been identified as an important component that contributes to the immunosuppressive properties of tumours [5,103]. Tumours with reduced lactic acid production developed significantly slower than control tumours and showed increased infiltration with IFN-γ-producing T and NK cells [5]. Alternatively, lactic acid prevented up-regulation of nuclear factor NFAT in T cells, resulting in diminished IFN-γ production [5]. Unlike effector T cells, Tregs have been shown to utilise extracellular lactate by converting it to pyruvate, demonstrating their ability to grow in the tumour microenvironment and participate in tumour immune evasion by inhibiting the activity of effector T cells [6,104].

Cholesterol is enriched in the tumour microenvironment, but its effect on TILs remains controversial. Cholesterol present in the tumour microenvironment was shown to inhibit the activity of CD8^+^ TILs [127]. Upon entry into tumours, CD8^+^ T cells acquire cholesterol, expressed high levels of several immune checkpoints, including PD-1, 2B4, TIM-3, and LAG-3, and become exhausted. Cholesterol induces immune checkpoint expression by increasing endoplasmic reticulum (ER) stress in T cells [127]. Consequently, the ER stress sensor XBP1 is activated and regulates PD-1 and 2B4 transcription. Inhibiting XBP1 or reducing cholesterol in CD8^+^ T cells effectively restores anti-tumour activity [127]. Furthermore, Yang et al. have reported that genetic ablation or pharmacological inhibition of ACAT1, a key cholesterol esterification enzyme [128], by increasing cholesterol inclusion in the plasma membrane, potentiates the proliferation and effector function of CD8^+^ T cells [129]. In addition, it was shown that inhibition of ACAT1 in combination therapy with anti-PD-1 antibodies improved efficacy in controlling tumour progression [129]. To reconcile these opposing observations, it can be proposed that, upon activation of antigen, T cells rapidly increase cholesterol synthesis through the increased transcriptional activity of SREBPs [130]. De novo synthesised cholesterol is then esterified by ACAT in the ER and stored in lipid droplets. Thus, ACAT acts as a negative regulator of T cell activation by limiting the cholesterol available for incorporation into membranes. Moreover, the proportion of cholesterol in the ER must be tightly controlled in order to promote cell homeostasis and the activity of ACAT participate in this regulation [131]. In the tumour microenvironment, however, the excessive amounts of cholesterol produced by tumour cells enter TILs’ cytoplasm, accumulate in the ER, and could overwhelm ACAT activity, thereby activating XBP1 and the transcription of inhibitory checkpoints.

## 7. Concluding Remarks

Numerous studies have shown that the regulation of immunity by T cells effectively controls the onset of disease and maintains immune homeostasis. Metabolic regulation is important for antigen-induced expansion and function of T lymphocytes. However, T cell metabolism is highly dynamic and T cells are able to operate in very different environmental conditions by adapting their metabolism. Although a large number of studies have reported the link between metabolic regulation and immune diseases, the idea of controlling T cell metabolism to provide precise immunotherapy is still in its infancy and will require extensive research to reach the patients that require it.
